# MmodalFire: A Continuous Multimodal Dataset Comprising Video and Physical Sensing Data for Detecting Indoor Fires

**DOI:** 10.1038/s41597-026-06810-6

**Published:** 2026-02-19

**Authors:** Yang Jia, Yihan Guo, Yetang Chen, Xinmeng Zhang, Gang Wang, Qixing Zhang

**Affiliations:** 1 Shaanxi Key Laboratory of Network Data Intelligent Processing, Xi’an University of Posts and Telecommunications, Xi’an, 710121 China; 2 School of Computer Science and Technology, Xi’an University of Posts and Telecommunications, Xi’an, 710121 China; 3 Xi’an CNNC Nuclear Instrument Co., Ltd, Xi’an, 710061 China; 4 Qixing Zhang is with the State Key Laboratory of Fire Science (SKLFS), the University of Science and Technology of China, Hefei, 230026 China

**Keywords:** Natural hazards, Electrical and electronic engineering

## Abstract

Because no multimodal dataset was previously available for fire detection research, we developed the MmodalFire multimodal fire detection dataset for training and evaluation of indoor fire detection algorithms. This publicly available dataset includes video and physical sensing data for fire detection use. The dataset comprises 65 videos that simultaneously captured six physical sensing data types, including smoke density, temperature, and infrared and ultraviolet radiation at 5 μm, 4.4 μm, and 3.8 μm. All data were acquired using monitoring cameras and fire sensors deployed as part of a fire detection system that was carefully designed to cover all possible variations, including different wind velocities, illumination conditions, common interference types, and occlusions. All videos and corresponding physical sensing data sequences are labeled as either fire or non-fire sequences. Using the MmodalFire dataset, we evaluated four basic baseline fusion models and the proposed dynamic fusion models to provide a reference for multimodal fire detection research under controlled laboratory settings, promoting research on multimodal fire detection algorithms using controlled-setting data.

## Background & Summary

To prevent large-scale fire and smoke damage, rapid and accurate fire detection is essential. Earlier detection increases the chances of survival of a fire. At present, construction codes in many countries require installation of spot-type detectors such as thermal and smoke detectors in all large buildings. These detectors are mostly installed on the ceilings in these buildings and generate an alarm when the heat or smoke reaches the detectors. However, during an actual fire, precious time can be lost before heat or smoke reaches the detectors to trigger the alarm, particularly in more spacious buildings. Because it combines rapid response times with a wide monitoring range and visibility, video fire detection (VFD) technology has become a hot topic in both research and industry^1–3^. VFD can respond to a fire’s physical characteristics much more rapidly than traditional spot-type detectors. When the camera captures a fire event, an alarm is generated after a very short reference period. However, VFD cannot respond to occluded fire images, and each sensing modality obviously has its own strengths and limitations.

Traditional fire detection systems mainly rely on unimodal detection, which cannot satisfy the requirements at important monitoring sites, such as thermal and nuclear power stations, supercomputer centers, and forests^4^. Rather than use only a single type of fire sensor, fusion of multiple sensing modalities for fire detection can be used to overcome the limitations of certain sensors by compensating using other sensors. Although various multimodal/multisensory fire detection methods have been proposed previously^5,6^, most multimodal detection methods use either nonvisual^7,8^ or visual^6^ approaches. In nonvisual fire detection, high temperatures, smoke, and the presence of carbon monoxide (CO) are typical fire characteristics that researchers have fused with backpropagation neural networks (BPNNs) to develop indoor fire warning systems^7,8^. Yang *et al*.^9^ fused the smoke density, CO concentration, and temperature characteristics with the residual currents in electrical lines to detect electrical fires. However, techniques that use a combination of video and physical sensing fire detection methods are uncommon. In the literature, we found only one publication related to integration of a nonvisual component (a fuzzy logic model that fuses the current and temperature information received from an electrical wire) with a visual component (implemented using a pretrained MobileNet model for two-dimensional (2D) fire detection) to identify the causes of electrical fires^10^. However, the two components were not fused to provide a complementary decision, unlike the multimodal fusion method that we present herein.

Most previous fire detection studies have been based on either nonvisual or visual fire features alone, and the use of a multimodal fire detection system that processes both physical fire features (nonvisual) and images (visual) is rare for several reasons. Most VFD researchers work in the computer vision (CV) field and tend to focus on video analysis, image processing, and deep-learning methods^3,11–13^. Multimodal analysis is different from CV, in which the sensors, the data acquisition systems, and heterogeneous data fusion must be considered. However, nonvisual analysis is related to sensors and unidimensional signal processing, and CV lies outside this research scope. With the rapid development of deep learning techniques, fire detection requires simultaneous processing of both VFD and physical sensing signals. Additionally, it is difficult to obtain sufficient training data for multimodal fire detection because the binary data that can be acquired from detectors lacks the valuable information required for analysis. Moreover, data collection for multimodal fire detection research is difficult.

To build a multimodal-based fire detection model, the data required must be captured simultaneously using multiple sensors. At present, however, no public dataset for fire detection in which both video and physical sensing data have been recorded simultaneously is available. Therefore, we developed a public multimodal fire detection dataset named MmodalFire, where the initial upper-case ‘M’ indicates multiple modalities. MmodalFire represents a fire scene at which multiple fire sensors are present and can be used to evaluate the effectiveness of fire recognition techniques for indoor scenes. The main contribution of this study is the compilation of data into a publicly available fire detection dataset that comprises simultaneously and continuously captured video and physical sensing data sequences to provide a reference for comparison of multimodal fire detection algorithms evaluated based on this dataset, as discussed in a previous study^14^.

The combination of video and physical sensing data for multimodal fire detection can also be used in many other research areas, including multimodal fusion strategies, co-learning, and transformer-based learning. Additionally, the MmodalFire dataset is designed to support the development and evaluation of multimodal fire detection algorithms under standardized laboratory conditions, including:

1. Training deep learning models integrating video and physical sensing data;
2. Comparing multimodal fusion strategies for controlled fire scenarios;
3. Studying fire behavior and sensor-video alignment in standardized settings.

**Fig. 1:**
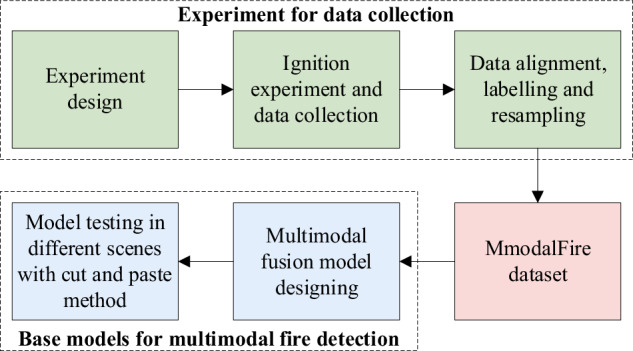
Workflow for multimodal fire detection dataset construction.

The workflow for the proposed method, which comprises acquisition of the experimental data to build the MmodalFire dataset and development of the multimodal fusion model, is shown in Fig. 1.

### Related works

For nonvisual fire detection, we did not find any public datasets composed of physical sensing data in the literature, possibly because of the limited authority available to obtain fire monitoring data from existing spacious buildings. Therefore, it has been necessary for researchers to build experimental platforms to collect sensor-based data on physical fire characteristics.

Multiple datasets for visual fire detection^15–19^, including VisFire^16^, the Keimyung University (KMU) Fire and Smoke Database^20^, the Video Smoke Detection (VSD) dataset^17,18^, etc, have been compiled in recent decades. Visual fire detection datasets usually comprise a combination of videos or images characterized by fire and other videos that do not contain any events of interest but specifically contain types of interference, including red objects moving in scenes, clouds, and the shadows of objects when illuminated by sunlight. Although recent research and development activity has led to more datasets containing smoke density or flame data being published, the dataset scale for a specific object detection scene is still very small, which usually includes dozens of videos, as shown in Table 1, when compared with the corresponding scale of the ImageNet^21^ and Common Objects in Context (COCO)^22^ datasets. In addition, combustion materials are selected randomly rather than being selected based on the standard of fire detection and fire alarm systems^23^, and the datasets comprise videos or images of building, industrial, and forest fires, and fires related to car accidents and riots. According to fire detector standards^23,24^, cotton, wood, n-heptane, and polyurethane, which represent the most common combustion materials used in building construction, should be considered. For example, curtains and bedclothes are often made from cotton, while furniture, doors, and floors are usually made from wood. In addition, indoor liquids such as alcohols, gasoline, and oils contain chemicals similar to n-heptane, and the stuffing materials used in sofas, toys, and mattresses are often made from polyurethane. It is thus necessary to establish an integrated fire detection dataset because of these detector standards^23,24^.

**Table 1:** Scales of related video fire detection datasets.

Dataset	Number of samples	Combustion materials
VisiFire^16^	30 videos	Forest, wood, kerosene
Huang’s dataset^17^	8885 images	N-heptane
BoWFireDataset^19^	466 images	All kinds of flame images collected from Internet
KMU Fire & Smoke Database^20^	38 videos	Gasline, heptane, wood
VSD Smoke Dataset^52^	30 videos/images <5000	Cotton rope, kerosene
Mivia Smoke Detection Dataset^15^	149 videos	Forest

Because no public datasets are currently available for multimodal fire detection, we designed a series of combustion experiments and collected the required physical sensing and video data simultaneously. All data were annotated as either fire or non-fire data.

Most recently proposed fire detection methods are video or image based fire detection methods using CNNs^17,18,25–34^ and transformers^35,36^, which can be regarded as a typical object detection or classification task. Although multimodal fire detection has been proposed^5,37^, most earlier studies initially focused on fusion of the smoke density, CO, temperature with simple rules^9,37–39^. The fusions merely integrate physical parameters, without involving visual data. The possible reason might be for the fusion research, a group of sensors need to be installed to capture the physical data and videos simultaneously. It is much more difficult than collecting images and videos from Internet or already installed surveillance system. Therefore, to support multimodal-sensing-based fire detection, we designed an experiment to build a multimodal dataset composed of video and physical-sensing data sequences. To the best of our knowledge, this is the first multimodal dataset to be developed for fire detection applications.

## Methods

To support multimodal fire detection, an experiment system was built to collect foundational data for the multimodal smoke–flame dataset. Smoke density, temperature, flame radiation and images were captured with different sensors simultaneously.

### Data collection system

Two laboratories are located in Xi’an and Hefei in China. The external ambient temperature was approximately 30 °C and the experimental room’s dimensions were 5 m × 3 m × 3 m. The background was fixed, and the experimental equipment included an elevating platform, a fire tray, an electric furnace, an igniter, a fan, a humidifier, detectors (for temperature, smoke, and flame radiation), and a 1080 P red–green–blue (RGB)–infrared video camera.

**Fig. 2:**
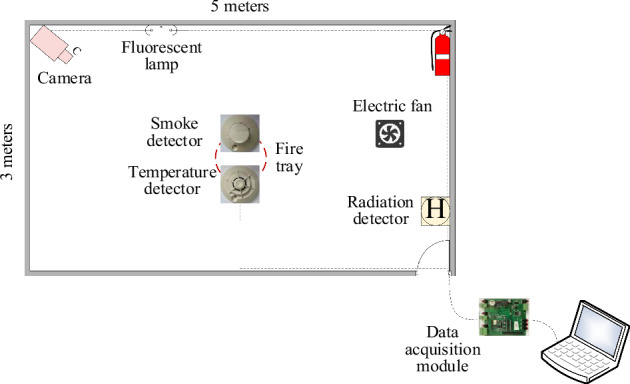
Schematic of experimental layout showing the smoke, temperature, and radiation detectors used to monitor three and one wavelength(s) in the infrared and ultraviolet bands, respectively.

The video camera had a resolution of 8 million pixels, a focal length of 2.8 mm, a night vision range of 30 m, and a frame rate of 30 frames per second (fps). The temperature and smoke detectors were manufactured according to Chinese standard^24^. The original data consisted of dimensionless numbers that were digitized and preprocessed by the detector. In addition, to capture the radiation values for the desired infrared and ultraviolet (UV) bands, a multiband pyroelectric infrared sensor that receives physical sensing data at 64 Hz was designed. Some of the detectors used in the experiment are shown in Fig. 2.

**Fig. 3:**
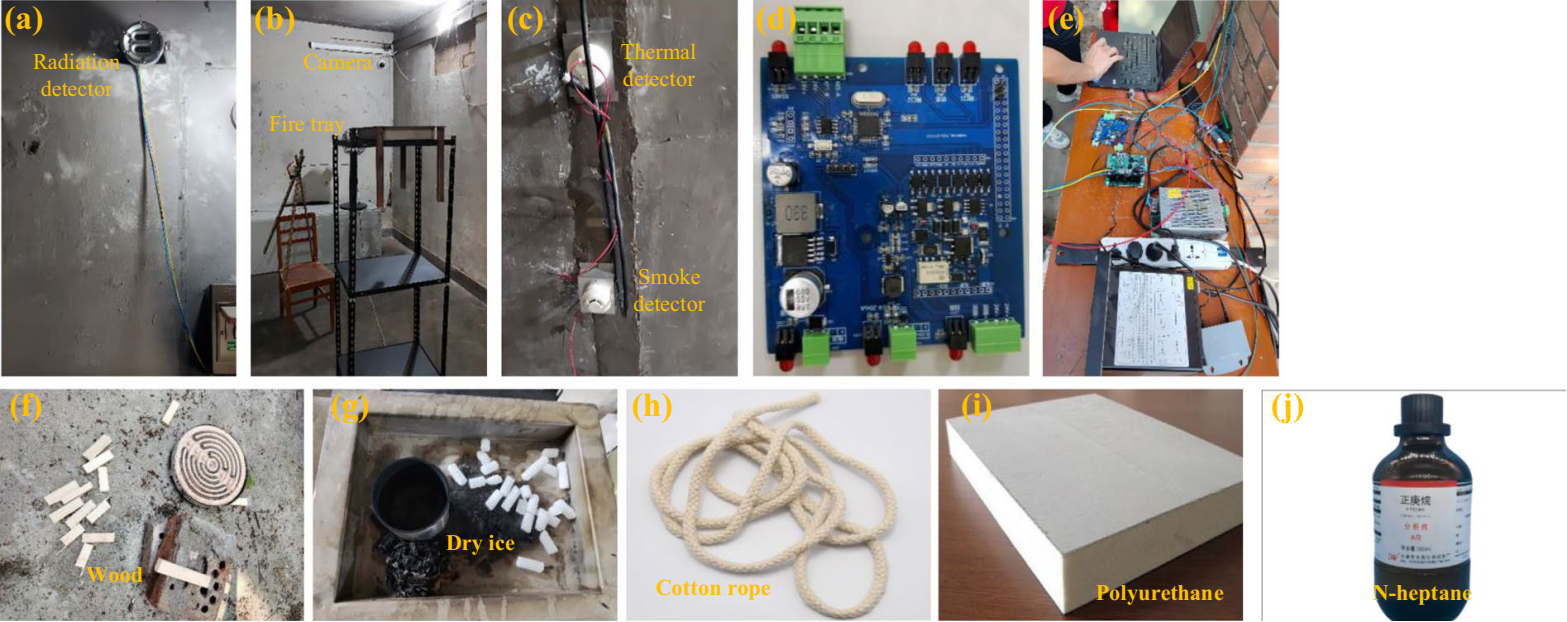
Experimental detectors and hardware: (**a**) Radiation detector integrated with infrared pyroelectric sensor and UV detector. (**b**) Video camera and fire tray. (**c**) Thermal and smoke detectors. (**d**) Homemade data collection board. (**e**) Connections for all the detectors and hardware. Experimental materials: (**f**) Wood pieces. (**g**) Dry ice. (**h**) Cotton rope. (**i**) Polyurethane. (**j**) *n*-heptane.

The experimental detectors and hardware are shown in Fig. 3(a–e). In one corner of the experimental room, the camera was installed near the ceiling to obtain a panoramic view of the fire scenes. The temperature and smoke sensors were installed at the center of the ceiling. In another corner of the room, the flame radiation detector was installed on the ceiling, diagonally across from the camera. Radiation data were collected at the 5.0 µm, 4.4 µm, and 3.8 μm infrared and UV wavelengths. Although the radiation values may be affected by both sunlight and human radiation, the radiation’s flame response is unique and it can therefore be used as a fire indicator. We also designed a module to collect all six physical sensing data streams from the serial ports and transmit these streams to a computer. Because we designed all the detectors, the data acquisition module, and the software specifically, the entire system is suitable for research and industrial applications.

### Four fire types and two interference types

Experimental materials including wood, dry ice, cotton rope, polyurethane, and n-heptane are shown in Fig. 3(f–i). There are four fire types and two interference types.

1. *Wood fire:* As per the experimental design, the wood was heated using a 2000 W electric furnace, as shown in Fig. 3(f). The wood ignited and then combusted in approximately 20 and 10 min, respectively. During early wood combustion, no flames and only a trace of smoke were observed. With continuous heating, the smoke density rose gradually. When the flame ignited, the wood then burned violently and the smoke density decreased slightly. During combustion, the temperature only changed negligibly, the smoke density increased, and the flame radiation was low.
2. *Cotton rope fire:* A 1.6-m-long cotton rope was placed on the fire tray, as shown in Fig. 3(h), and lit directly using the igniter. From ignition to extinction, the rope combusted relatively rapidly but less intensely than the wood, and less smoke was generated in this case. With the gradually increasing smoke density, the flame was small, and only limited heat was generated. During combustion, the temperature changed negligibly, the smoke density increased slowly, and the flame radiation was low.
3. *Polyurethane foam fire:* Polyurethane foam was placed on the fire tray, as shown in Fig. 3(i), and a tinfoil layer was placed below the foam to protect the fire tray and ignite the foam directly. Because polyurethane is an organic polymer, considerable black smoke containing toxic gases was generated during combustion. The flame was large, generating substantial heat, and the room’s temperature rose substantially. During combustion, the temperature increased rapidly, the smoke density increased sharply, and the flame radiation was high.
4. *n-Heptane fire*: In each trial, 60 mL of *n*-heptane was placed in an iron container and lit paper was used to ignite the liquid (Fig. 3(j)). When compared with polyurethane, *n*-heptane is purer, containing only carbon and hydrogen, and produces a clearer flame and negligible smoke. The *n*-heptane flame was observed easily. During combustion, although almost no smoke was generated, substantial heat was generated, the room’s temperature increased slowly, the smoke density change was negligible, and the flame radiation was high.
5. *Dry ice:* Stage smoke was simulated using dry ice (Fig. 3(g)). At room temperature (35 °C), the dry ice surface absorbs moisture hygroscopically from the air to form an ice film that impedes direct contact between the dry ice and air and slows water mist generation. Therefore, hot water was added to the fire tray to generate water mist rapidly. Figure 4(e1,e2) show examples of the dry-ice-derived water mist that were generated without and with hot water, respectively. During the smoke simulation, the changes in the temperature, smoke, and radiation detector outputs were negligible;

**Fig. 4:**
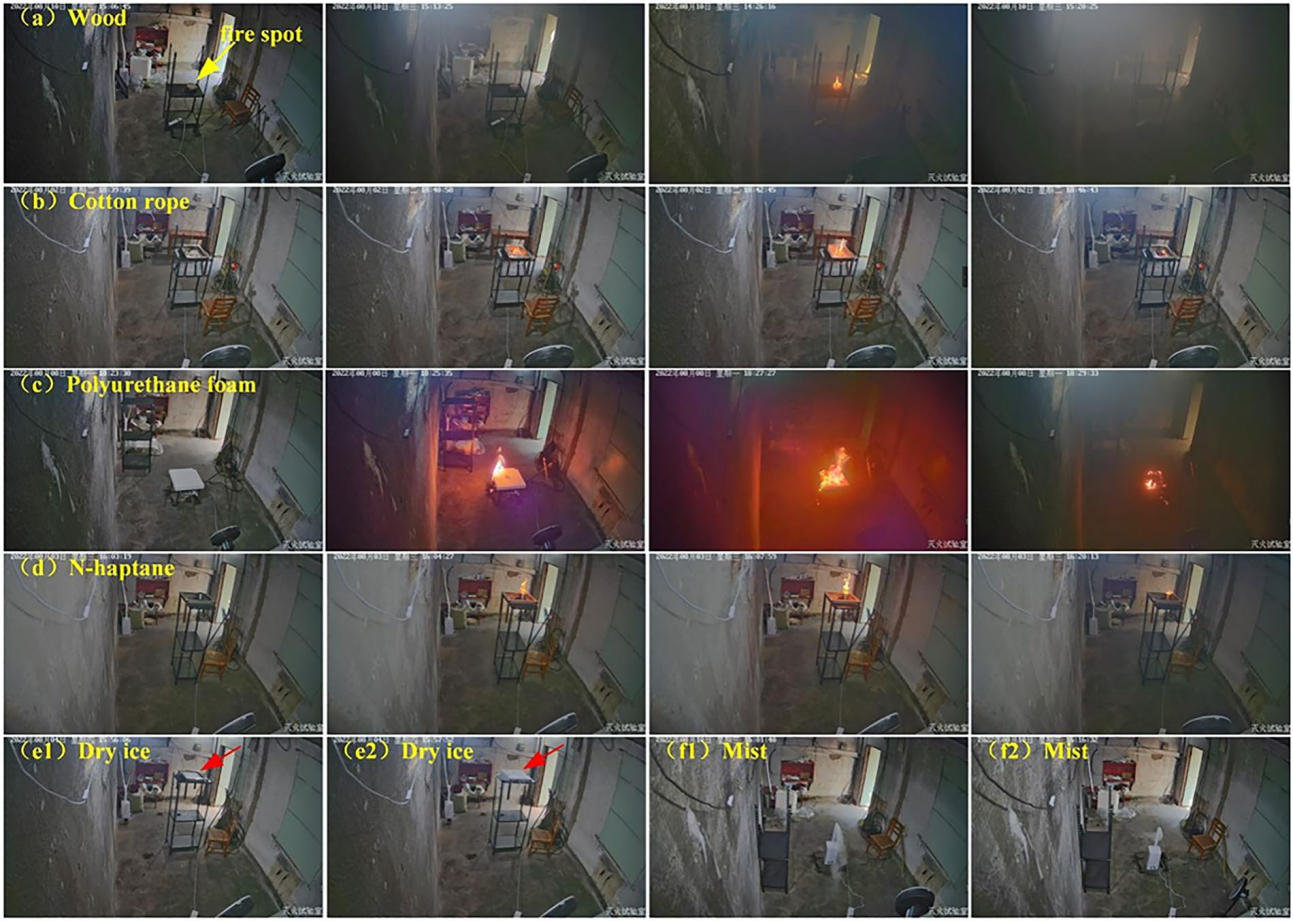
Example video frames. (**a**–**d**) are four fire types. In (**a–d**), for each row, there are 4 frames sampled from one video according to the time sequence. (**e**) and (**f**) are two negative samples of dry ice and mist.
6. *Water mist:* Water mist was simulated using a humidifier. When the humidifier is on, it is difficult to distinguish between actual smoke and the humidifier-generated water mist. Figure 4(f1,f2) show the water mist when sprayed using the humidifier before and after the fan was turned on, respectively. During the water mist simulation, the temperature, smoke, and radiation detector output changes were negligible.

### Combustion experiment settings for data collection

For the 34 different experimental control groups, the luminance, wind velocity, fire location, and material quantity values are listed in Table 2.

**Table 2:** Combustion experiment settings for data collection.

Control Group No.	Fire/Interference	Combustion material	Luminance (lx)	Wind velocity(m/s)	Location of fire	Quantity of material
1	Fire	Cotton 1	168	0	Close to the wall, 1.2 m to the ceiling	3.2 m to the ceiling
2		Cotton 2	34	1.0–1.5	Close to the wall, 1.2 m to the ceiling	4.8 m
3		Cotton 3	96	0	Center of the room, 1.8 m to the ceiling	1.6 m
4		Cotton 4	98	0.5–1.0	Center of the room, 1.8 m to the ceiling	1.6 m
5		Cotton 5	35	0	Center of the room, 1.8 m to the ceiling	1.6 m
6		Cotton 6	39	0.5–1.0	Center of the room, 1.8 m to the ceiling	1.6 m
7		Cotton 7	148	0	Center of the room, 1.2 m to the ceiling	1.6 m
8		Cotton 8	70	0	Close to the detector, 1.2 m to the ceiling	1.6 m
9		Cotton 9	150	0	Center of the room, 1.2 m to the ceiling	3.2 m
10		n-Heptane 1	180	0	Center of the room, 1.2 m to the ceiling	75 ml
11		n-Heptane 2	180	1.0–1.5	Center of the room, 1.2 m to the ceiling	75 ml
12		n-Heptane 3	50	0	Center of the room, 1.2 m to the ceiling	75 ml
13		n-Heptane 4	50	1.0–1.5	Center of the room, 1.2 m to the ceiling	75 ml
14		n-Heptane 5	65	0	Close to the detector, 1.2 m to the ceiling	75 ml
15		n-Heptane 6	65	0	Close to the detector, 1.2 m to the ceiling	75 ml
16		n-Heptane 7	172	0	Close to the wall, 1.2 m to the ceiling	75 ml
17		n-Heptane 8	59	0	Close to the wall, 1.2 m to the ceiling	75 ml
18		Polyurethane 1	159	0	Center of the room, 1.2 m to the ceiling	3 pieces, 50*50*3 (cm)
19		Polyurethane 2	62	0	Center of the room, 2.8 m to the ceiling	2 pieces, 50*50*3 (cm)
20		Polyurethane 3	48	0.5–1.0	Center of the room, 2.8 m to the ceiling	2 pieces, 50*50*3 (cm)
21		Polyurethane 4	20	0	Center of the room, 2.8 m to the ceiling	2 pieces, 50*50*3 (cm)
22		Polyurethane 5	20	1.0–1.5	Center of the room, 2.8 m to the ceiling	2 pieces, 50*50*3 (cm)
23		Wood1	61	0	Center of the room, 1.8 m to the ceiling	2 pieces, 2.5*2.5*10 (cm)
24		Wood2	61	1.0–1.5	Center of the room, 1.8 m to the ceiling	2 pieces, 2.5*2.5*10 (cm)
25		Wood3	44	0.5–1.0	Center of the room, 1.0 m to the ceiling	2 pieces, 2.5*2.5*10 (cm)
26		Wood4	21	0	Center of the room, 1.0 m to the ceiling	2 pieces, 2.5*2.5*10 (cm)
27	Interference	Dry ice1	106	0.5–1.0	Close to the wall, 1.2 m to the ceiling	20 pieces, (no hot water)
28		Dry ice2	106	0.5–1.0	Close to the wall, 1.2 m to the ceiling	20 pieces, (with hot water)
29		Dry ice3	137	0	Close to the wall, 1.2 m to the ceiling	20 pieces, (with hot water)
30		Dry ice4	47	0	Close to the wall, 1.2 m to the ceiling	20 pieces, (with hot water)
31		Mist1	84	0	Center of the room, 2.8 m to the ceiling	—
32		Mist2	84	1.0–1.5	Center of the room, 2.8 m to the ceiling	—
33		Mist3	30	1.0–1.5	Center of the room, 1.2 m to the ceiling	—
34		Mist4	30	0	Center of the room, 1.2 m to the ceiling	—

The combustion material, luminance, wind velocity, fire location, and interference are controlled to ensure the diversity of the dataset. Details are as follows. (1) **Combustion material selection:** The fire experiment was designed based on the national standard for the People’s Republic of China^53^ and most settings used were consistent with those in the corresponding British standard (BS EN 54)^23^. Four main combustion materials were used, including several pieces of 10 × 2 × 3 cm^3^ beechwood batten, eight pieces of 50 × 50 × 6 cm^3^ polyurethane sponge, a 1-cm-diameter cotton rope, and 800 mL of *n*-heptane (>99% purity). (2) **Lighting environment:** The light intensity was divided into strong and weak ranges (>100 and 0–100 lux) that corresponded to the intensities of indoor lighting on sunny and cloudy days, respectively. (3) **Wind Velocity:** The airflow was divided into strong, light, and no wind ranges (1.0–1.5, 0.5–1.0, and 0 m/s, respectively) that were used to simulate common natural wind conditions. (4) **Occlusion:** The camera was either occluded completely using a black cloth to eliminate video monitoring or was semi-occluded by changing the fire’s location to limit the view that the camera could capture. (5) **Sunlight and pedestrian radiation:** The detected radiation levels varied because of sunlight and human activity-derived radiation. (6) **Environmental interference:** For the negative samples, the smoke that would be produced in fires was simulated using dry ice and a humidifier to generate steam and water mist, respectively. The other environmental variables remained constant during each experiment.

To increase the diversity of the dataset and alleviate generalization limitations, we conducted 2 sets of experiments in 2 laboratories with different scenario settings. The dataset is designed for the research of multi-institution research and model generalizability.

### Annotations

#### Time alignment

Because every video frame and the data streams that were captured using the other sensors had unique time stamps at a resolution of 0.01 s, the video and physical sensing data can be used either separately or simultaneously for any time stamp.

#### Labeling

Because the proposed dataset uses binary classification (i.e., fire or non-fire), the data for the four fires and the two interference types were labeled fire and non-fire, respectively.

## Data Records

The dataset is available for download from Figshare^40^, comprising two file folders (MmodalFire (Xi’an) and MmodalFire (Hefei)), which compile the captured sensor data from two laboratories in this study. In one folder, there are two folders (InertialData and VideoData) and a worksheet. The contents of the worksheet and the meaning of its columns are detailed in Table 3.

**Table 3:** Description of the settings of the combustion experiment for data collection (for the file “SETTINGS OF THE COMBUSTION EXPERIMENT FOR DATA COLLECTION.xlsx”).

Sheet	Column	Description
SETTINGS OF THE COMBUSTION EXPERIMENT FOR DATA COLLECTION.xlsx	File name(.csv,.mp4)	File names of the sensor data and videos in the folders “InertialData” and “VideoData”
	Material	Types of combustion material
	Lighting Environment(lx)	Light intensity of the laboratory
	Wind speed (m/s)	Air flow rate of the laboratory
	Location of fire	Related to the distance between fire and detectors
	Quantity of material	—

In the folder “InertialData”, the sensor output data is recorded in each.csv files, such as “sub1_trx.csv”. In each file, there are 6 types of physical sensing data, including the smoke density, temperature, infrared radiation at three different wavelengths, and UV radiation, were measured and processed as dimensionless sensor outputs. The minimum, maximum, and typical values that were measured without and during a fire are also listed in Table 4.

**Table 4:** Detailed description of the physical data.

Sheet	Column		Description				
			Minimum value	Maximum Value	Typical value without fire	Typical value during fire	Characteristics of the value
sub1_trx.csv	Smoke density		800	3400	800–1400	1401–3400	The larger the fire, the greater the numerical value
	Temperature		1000	2700	1000–1500	1501–2700	
	Radiation	5 μm	300	4100	2600–2800	300–4100	Under normal conditions, the values fluctuate gently within a small range, while during a fire, the values fluctuate violently over a large range
		4.4 μm	0	4100	2250	0–4100	
		3.8 μm	0	4100	2100–2400	0–4100	
		ultraviolet	1	400	1	2–400	

Typical physical sensing data of smoke density, temperature, and radiation values.

*Because all data has been preprocessed in sensors, the sensor output is dimensionless.

Example video frames for four fire types are shown in Fig. 4(a–d), with four frames that were clipped at different time points in each fire’s development, and Fig. 4(e,f) show example frames for both interference types.

## Technical Validation

### Data preparation for fire detection

Before the original multimodal data were used to perform fire detection, the data were processed as follows.

**Fig. 5:**
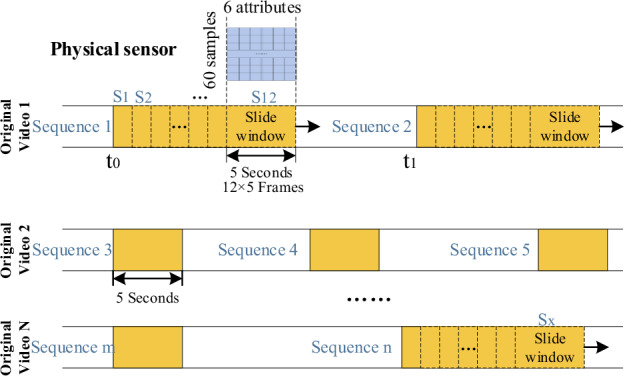
Explanation of data sampling process. For each experiment, a total of 65 long videos lasting more than 45 s were recorded, from which group of 5-seconds long nonoverlapping sequences were clipped. For a positive control video (i.e., recorded during a fire), at least two nonoverlapping sequences were clipped manually. For a negative control video (i.e., recorded without a fire), at least one sequence was clipped manually. A one-frame sliding window increment was used for each sequence, and 11 subsequences were generated from each original sequence. Therefore, each yellow block represents a total of 12 subsequences. The blue block illustrates the data recorded for 60 samples using six sensors in 5 seconds.

Sixty-five videos were recorded during combustion of the different materials, as shown in Table 2. Each video was approximately 45 s long and the training dataset comprised nonoverlapping 5 s sequences with sliding window (step = 1 frame). As shown in Fig. 5, each yellow block represents a 5 s sequence. For a positive control video (recorded during a fire), at least two nonoverlapping sequences were clipped manually. For a negative control video (recorded without fire), at least one sequence was clipped manually.

Because the sampling frequencies differed for all the detectors, the acquired data had to be aligned before processing. We aligned the data sequences by searching for the best local alignment of a short (5-s-long) segment. (The temperature and smoke detector data were sampled 64 times per second, and the update interval was 4 s. Therefore, within a 4 s period, all 64 outputs were identical. To obtain at least two different values, the multimodal data cycle alignment was set at 5 s, i.e., longer than 4 s.) Because all videos were recorded at 12 fps, 5 s of video comprised a total of (12 × 5 = ) 60 frames, as shown in Fig. 5. Additionally, in 5 s, a total of (64 × 5 = ) 320 lines of multisensor data were recorded. Downsampling was thus used to reduce the number of multisensor data and 60 lines were finally selected for use as training data. To process the unidimensional data obtained from the sensors, we arranged the six physical sensing data types into a 2D array with six columns and 60 rows, as illustrated by the blue block in Fig. 5. Therefore, one input training datum comprises 60 frames and 60 lines of multisensor data in 5 s.

A convolutional network was designed to train using multiple data subsets, and the resulting model was validated and tested using data acquired from other subsets to predict current regional fires.

To validate the usability of the MmodalFire dataset and provide basic reference baselines for researchers using this dataset, we implemented four simple baseline models and a reference fusion model.

**Fig. 6:**
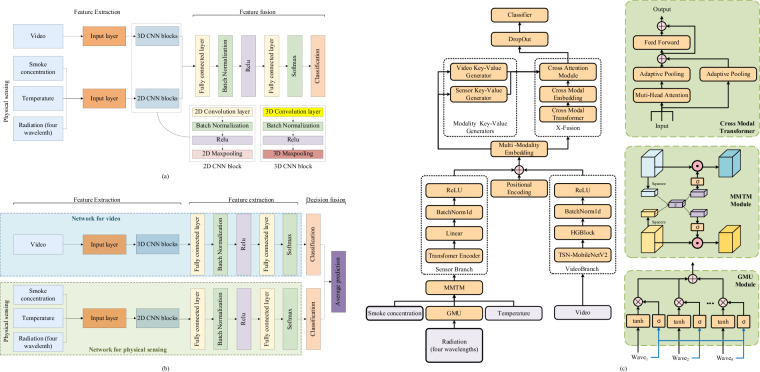
Architecture of the reference fusion model for dataset usage, demonstrating a typical workflow for fusing video and sensor data from the MmodalFire dataset. (**a,b**) are two baseline models, and (**c**) is the reference fusion model. The video and sensor branch independently process video and physical sensor data. In the sensor branch, the GMU module first integrates data from four different wavelength sensors, followed by the MMTM module which combines smoke concentration, temperature, and wavelength features before passing the output into the transformer encoder. In the video branch, TSN-MobileNetV2 and HGBlock are used to process the video data. The X-Fusion module is responsible for fusing the outputs from both the sensor and video branches.

The study environment was composed as follows. The operating system was Ubuntu 18.04.1, the central processing unit (CPU) was the Intel i5-10400, the graphics processing unit (GPU) was the NVIDIA RTX 2080, and the deep learning framework was PyTorch. We used two common modality fusion models and the reference fusion model (FM) to evaluate the fire detection accuracy. The optimized model parameters are as follows: For the single modality (physical sensing) model, the batch size is 200, the number of epochs is 100, the dropout rate is 0.5, and the learning rate is 0.004. And for the FM, the batch size is 32, the number of epochs is 10, the dropout rate is 0.5, and the learning rate is 0.004. The flowchart for the feature fusion network is shown in Fig. 6. The video and sensor branch independently process video and physical sensor data. In the sensor branch, the GMU module first integrates data from four different wavelength sensors, followed by the MMTM module which combines smoke concentration, temperature, and wavelength features before passing the output into the transformer encoder. In the video branch, TSN-MobileNetV2 and HGBlock are used to process the video data. The X-Fusion module is responsible for fusing the outputs from both the sensor and video branches.

#### Sensor branch

To fuse radiation data from four wavelengths, we adopt a Gated Multimodal Unit (GMU)^41^ as a reference method. The core fusion formula is as follows:

1 $$h={\sum }_{i=1}^{4}{z}_{i}\odot {h}_{i}.$$

where the gating vector components *z*_*i*_ (from concatenated wavelength features) are computed via the sigmoid function σ(⋅), and the modality-specific features *h*_*i*_ are obtained via the tanh function. This fusion approach adaptively balances the contribution of each wavelength feature, serving as a basic reference for using the dataset.

To demonstrate the dataset’s support for fusing multimodal sensor features (smoke concentration, radiation, temperature), we adopt the Multimodal Transfer Module (MMTM)^42^ as a reference fusion method in the sensor branch. This module uses a “squeeze-and-excitation” style mechanism to adjust feature contributions across modalities, serving as a typical example of using the dataset for multimodal fusion tasks.

In the reference architecture, the three sensor modalities are processed via separate embedding branches to obtain feature vectors *f*_*1*_, *f*_*2*_, *f*_*3*_. We integrate MMTM between the feature encoders and classifier to recalibrate inter-modal features; the core process involves:

1. Deriving a shared latent vector from concatenated features (via linear transform);
2. Computing modality-specific excitation vectors;
3. Recalibrating original features via sigmoid gating.

The final fused representation *X*_*fused*_ (fed to downstream layers) is given by:

2 $${X}_{{\rm{fused}}}=[\sigma ({e}_{1})\odot {f}_{1},\sigma ({e}_{2})\odot {f}_{2},\sigma ({e}_{3})\odot {f}_{3}]$$

where σ(⋅) denotes the sigmoid function, ⊙ denotes element-wise multiplication, and [⋅] signifies feature concatenation.

After the sensor fusion module, the feature tensor output by MMTM is fed into a Transformer encoder. We adopt this Transformer-based temporal encoder^43^ (instead of a recurrent network) to process the time-series inertial sensor data in the MmodalFire dataset, demonstrating the dataset’s support for extracting temporal dependencies from sequential sensor signals.

#### Video branch

In the video branch, the features are passed through a TSN-MobileNetV2 block^44^, which leverages a MobileNetV2 backbone integrated with a Temporal Shift Network(TSN) mechanism. MobileNetV2 is a lightweight, efficient convolutional network, while TSN allows for efficient processing of video sequences by modelling temporal dependencies. The combined architecture extracts both sptial and temporal features from video frames.

The output from TSN-MobileNetV2 is then passed through an HGBlock^45^ for enhanced feature extraction. This block further refines the extracted features, enabling the model to focus on relevant video content and discard unnecessary noise. The final feature representation is output from the Video Branch, ready for fusion with sensor modalities or further processing in the classification head.

#### Fusion module for the video branch and sensor branch

To demonstrate the MmodalFire dataset’s support for fusing video and sensor branch features, we adopt the X-Fusion block^45^ as a reference method. This module is used to illustrate how the dataset’s multimodal data (video + physical sensing) can be integrated for fire detection tasks, with details simplified here to focus on the dataset’s applicability.

In the reference workflow, the sensor branch feature s and video branch feature v (derived from the dataset) are first projected into a unified dimension. The projected features are then processed via a cross-modal Transformer to enable inter-modal interaction, and key-value pairs are generated to refine feature alignment. The final fused feature vector (fed to the classifier) is:

3 $${f}_{{fused}}=[{h}_{{sensor}},{h}_{{video}}]\in {{\mathbb{R}}}^{2D}$$

where *h*_*sensor*_ and *h*_*video*_ denote the recalibrated features of the two branches, and D is the unified feature dimension.This reference fusion process shows that the MmodalFire dataset can support complex multimodal integration workflows.

### Evaluation protocols

For a given test set, we used the accuracy, the precision, the recall, and the F-measure as the common quantitative indicators to evaluate the fire detection models, where each sample comprised a 5-s-long data sequence, as follows:

- True positive (TP): samples labeled as ‘fire’ were correctly classified as containing fire events.
- False negative (FN): samples labeled as ‘fire’ were incorrectly classified as containing no fire events.
- False positive (FP): samples labeled as ‘non-fire’ were incorrectly classified as containing fire events.
- True negative (TN): samples labeled as ‘non-fire’ were correctly classified as containing no fire events.

Threshold: When the predicted fire probability was above the threshold, a fire was occurring; otherwise, no fire was occurring.

*(1) Accuracy (of fire prediction):* the ratio of the number of all correct fire predictions to the number of test samples, as shown in Eq. (4):

4 $$Accuracy=(TP+TN)/(TP+TF+FP+FN)$$

*(2) Precision (of fire prediction):* the ratio of the number of all correct fire predictions to the number of samples predicted as ‘fire’, as shown in Eq. (5):

5 $$Precision=TP/(TP+FP)$$

*(3) Recall (of fire prediction):* the ratio of the number of all correct fire predictions to the number of ‘fire’-labeled samples, as shown in Eq. (6):

6 $$Recall=TP/(TP+FN)$$

*(4) F-measure:* the harmonic mean of the precision and recall, as shown in Eq. (7):

7 $$F-measure=\frac{2\ast Presicion\ast Recall}{Precision+Recall}$$

*(5) Average fire prediction probability:* the average fire prediction probability for all ‘fire’-labeled samples is shown in Eq. (8). Here, *P*_*f*1_ (1 < i < n) is the probability of the samples being predicted as ‘fire’ samples, and n is the total number of samples labeled as ‘fire’. This metric indicates the degree of fire prediction accuracy.

8 $$A{P}_{fire}=({P}_{f1}+{P}_{f2}+\mathrm{\ldots \ldots }+{P}_{fn})/n$$

*(6) Average non-fire prediction probability:* the average non-fire prediction probability for all ‘non-fire’-labeled samples is shown in Eq. (9). Here, ${P}_{nfi}$ (1 < i < m) is the probability of the samples being predicted as ‘non-fire’ samples, and m is the total number of samples labeled as ‘non-fire’.

9 $$A{P}_{non-fire}=({P}_{nf1}+{P}_{nf2}+\mathrm{\ldots \ldots }+{P}_{nfm})/m$$

### Experiment and results

The training-testing dataset splits are shown in Table 5, and the original dataset is shown in Table 2. A total of 65 original data groups were recorded, where 33 of these groups covered all the materials and were then selected as the training set. In each group, the training data were selected randomly, and the test set comprised pristine data selected from the remaining data. To clarify, the training-test split was conducted by random sampling across the two laboratories. Due to the core contribution being the dataset itself, this split is intended to provide a basic reference for algorithm usage, rather than a strict evaluation of cross-scenario generalization. A more rigorous protocol (e.g., train on Lab 1 and test on Lab 2) could be adopted in future work to further validate model robustness and avoid potential sample correlation.

**Table 5:** Experimental Training–Test Dataset Splits.

Original video groups of different combustion material	Total number of (group/sequence)	Training set (group/sequence)	Testing set (group/sequence)
Cotton	17/476	9/252	8/224
n-Heptane	14/392	7/196	7/196
Polyurethane	10/280	5/140	5/140
Wood	8/224	4/112	4/112
Dry ice	8/224	4/112	4/112
Mist	8/224	4/112	4/112
Total	65/1820	33/924	32/896

Two single-modality and four multimodality models were evaluated at different thresholds that indicated the approximate detection reliabilities used to generate a stable alarm. For example, at a threshold of 0.9, all predictions with values above 0.9 were treated as correct fire detections and produced either a TP or a TN.

#### Single-modality-based fire detection results

**Fig. 7:**
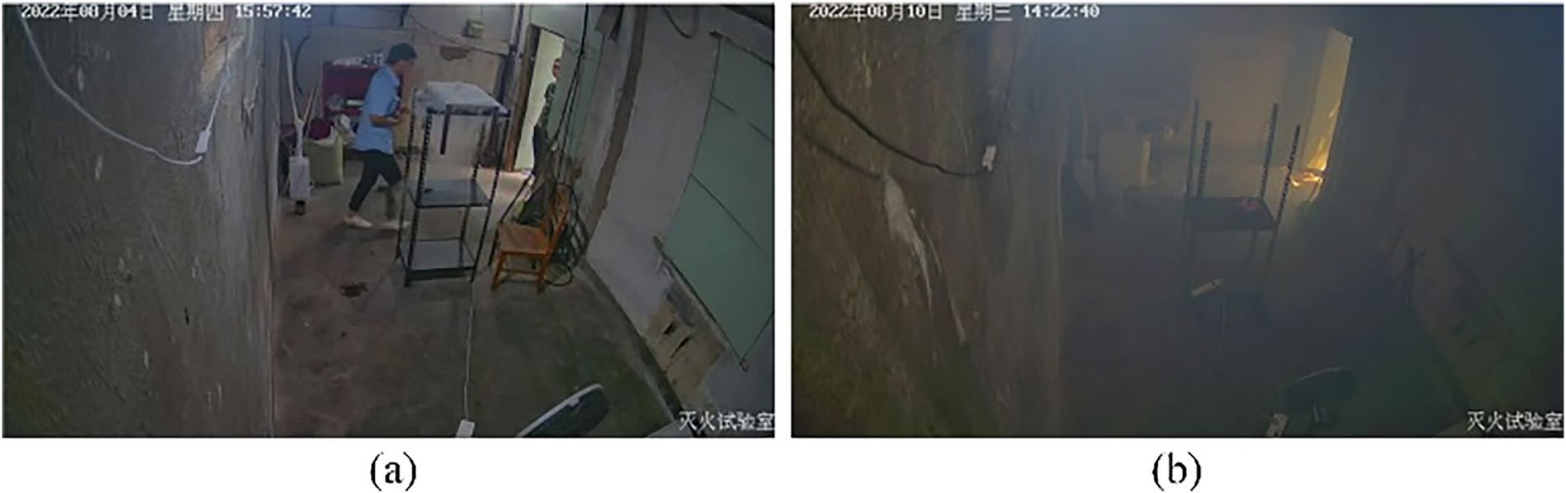
Examples of fire detection failures under specific conditions: (**a**) dry ice and pedestrian movement, and (**b**) a room full of smoldering-derived smoke during early wood burning.

In single-modality-based fire detection using either a video camera or a sensor, fire detection failed under some circumstances, as illustrated in Fig. 7. The failure details were analyzed as follows.

##### Failure of video-based fire detection

For the test data involving dry ice and pedestrian movement for 30 s, the video-based fire detection failed when the fire and non-fire thresholds were both set at 0.5. (If the predicted fire probability is more than 0.5, a fire is occurring; otherwise, no fire is occurring. However, if the predicted non-fire probability is more than 0.5, a non-fire event is occurring; otherwise, no fire is occurring.) In the scene shown in Fig. 7(a), although the predicted fire probability was 0.58, no fire was occurring. Because a person was walking in the room and the appearance of the dry ice was similar to that of real smoke, a false alarm was generated. However, in this situation, the other sensors functioned well and the predicted fire probability was 0.03. Within 30 s, the four fusion models predicted correctly that no fire was occurring.

##### Failure of physical-sensing-based fire detection

**Fig. 8:**
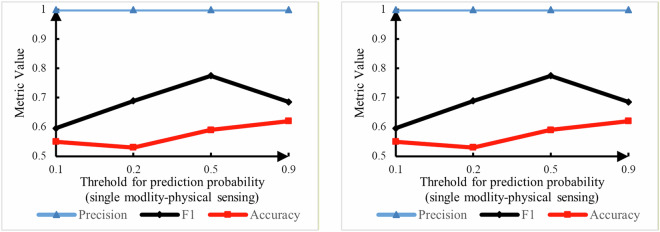
(**a**) video- and (**b**) physical sensing-based single-modality fire detection results.

The test set comprised data from a room full of smoldering-derived smoke during the first 30 s of wood burning, as shown in Fig. 7(b). As illustrated in Fig. 8(a), when the sensors malfunctioned, the FM models still functioned well when using only the video camera. APfire decreased by 0.01 to 0.05, and the change in APnon-fire was negligible. At this experimental threshold, the metrics did not change, and the FM models still predicted the fire accurately.

As shown in Fig. 8, for this data sample, the fire prediction results obtained using the physical sensing model were incorrect, even when the threshold was as low as 0.1, whereas the prediction accuracies of the other models were 1.0 at a threshold of 0.9 because, when the fire’s starting point was sufficiently far away from the ceiling and the smoke density was low, the smoke plume could not reach the ceiling quickly. Therefore, because the temperature and flame radiation values did not change and the smoke detector could not capture the changes in the smoke density, the physical sensing model output the incorrect detection result that no fire was occurring in the room.

#### Fusion Module Experiments

In addition to simple feature concatenation, we compare several representative multimodal fusion mechanisms:

1. Transformer Encoder^46^, standard self-attention structure applied to each modality and/or their concatenated features.
2. MMTM (Multimodal Transfer Module)^42^, which uses squeeze-and-excitation style channel-wise recalibration across modalities.
3. XFusionBlock^45^, a cross-modal Transformer-based block designed to support flexible combinations of modalities.
4. MFT (Multimodal Fusion Transformer)^47^, where tokens from each modality attend to each other via multi-head attention and then are globally pooled for classification.
5. Cross-attention fusion^48^, a Transformer-style block in which inertial and video tokens attend to each other via multi-head attention and are then globally pooled for classification.
6. Bilinear fusion^49^, which captures multiplicative interactions between modalities (e.g., outer-product or compact bilinear pooling) and enables richer cross-modal feature coupling.

These fusion variants are evaluated under the same backbone encoders to disentangle the effect of backbone capacity from fusion design.

In our work, we demonstrate how the MmodalFire dataset supports fusing three heterogeneous sensor modalities (smoke concentration, radiation, and temperature) for the sensor branch, using various fusion modules to illustrate the dataset’s applicability to multimodal fusion tasks.

Table 6 presents the performance of different fusion modules on the sensor branch, provided as basic references for researchers using the MmodalFire dataset. All results are obtained under consistent experimental settings to ensure fair comparison. MMTM is adopted in the reference model for subsequent experiments, and researchers can select fusion methods based on their specific task requirements.

**Table 6:** Comparison of Fusion Modules for Sensor Branch.

Model	Accuracy	Precistion	Recall	F1	Model size(MB)	GFLOPs
CNN + Concat(Baseline)^54^	0.837	0.831	0.745	0.786	25.04	11.915
Transformer Encoder^46^	0.874	0.877	0.832	0.854	25.28	11.938
MMTM^42^	0.932	0.943	0.957	0.950	25.55	11.939
XFusionBlock^45^	0.856	0.832	0.877	0.854	25.39	11.955
MFT^47^	0.732	0.91	0.938	0.924	25.29	11.935
FiLM^55^	0.793	0.75	0.875	0.762	25.36	11.938
Cross-attention^48^	0.854	0.832	0.844	0.838	25.62	11.939
Bilinear^49^	0.953	0.936	0.873	0.903	25.31	11.938

Different fusion modules exhibit distinct characteristics when applied to the dataset: Feature concatenation (baseline CNN + Concat) processes modalities without channel-level modulation; Transformer-based schemes (e.g., standard encoder or cross-attention) capture token/modality-level interactions; FiLM introduces feature-wise linear modulation; bilinear pooling captures multiplicative interactions. Each method aligns with different design priorities, and the dataset can accommodate these diverse fusion strategies.

These results illustrate that the MmodalFire dataset can support the evaluation of various fusion modules (each with distinct design tradeoffs) for sensor modality fusion tasks, providing flexible options for researchers using the dataset.

In addition, we conducted a set of experiments for the sensor + video branch (with fixed backbone encoders for both modalities, only swapping the fusion module) to demonstrate how the MmodalFire dataset supports heterogeneous modality fusion tasks. The results are summarized in Table 7.

**Table 7:** Comparison of Fusion Modules for Sensor and Video Branches.

Model	Accuracy	Precistion	Recall	F1	Model size(MB)	GFLOPs
CNN + Concat(Baseline)^54^	0.837	0.831	0.745	0.786	25.04	11.915
Transformer Encoder^46^	0.902	0.884	0.847	0.865	25.41	11.946
MMTM^42^	0.934	0.927	0.936	0.931	25.54	11.938
XFusionBlock^45^	**0.946**	**0.932**	**0.977**	**0.953**	25.39	11.953
MFT^47^	0.832	0.927	0.942	0.934	25.30	11.938
FiLM^55^	0.843	0.837	0.888	0.861	25.39	11.941
Cross-attention^48^	0.924	0.859	0.876	0.867	25.57	11.939
Bilinear^49^	0.913	0.900	0.894	0.896	25.33	11.940

Table 7 presents the performance of various fusion modules for the combined sensor + video branches, serving as a reference for researchers using the MmodalFire dataset. All results are obtained under consistent experimental settings (fixed backbones) to ensure the outcomes reflect the dataset’s compatibility with different fusion strategies. XFusionBlock is adopted in the reference model to illustrate the dataset’s ability to support flexible, token-level interaction between heterogeneous modalities (video and sensor data).

The use of XFusionBlock in the reference workflow shows that the dataset can accommodate fusion modules designed for cross-modal alignment—this example demonstrates how video and sensor data from the dataset can be integrated to support fire detection tasks. All results are reproducible with the provided dataset and code, and researchers can explore other fusion modules based on their specific task priorities (e.g., model size, interaction type).

These results do not represent the superiority of any fusion module; instead, they verify that the MmodalFire dataset can support the evaluation of diverse fusion strategies for heterogeneous (video + sensor) modality tasks. The result is shown in Table 8.

**Table 8:** Multimodality Fire Detection Results Obtained Using the Baseline Detection Models.

Model	Threshold for prediction probability	Accuracy	Precision	Recall	F-measure	Model size (MB)	GFLOPs
Baseline Model	0.1	0.842	0.892	0.834	0.862	25.04	11.915
	0.2	0.819	0.883	0.835	0.858		
	0.5	0.837	0.865	0.859	0.861		
	0.9	0.874	0.871	0.733	0.796		
Proposed FM	0.1	0.932	0.943	0.961	0.952	26.2	12.2
	0.2	0.947	0.932	0.955	0.943		
	0.5	0.97	0.943	0.974	0.958		
	0.9	0.976	0.911	0.988	0.947		

#### FM fire detection results

Table 9 presents the results of multimodal fire detection obtained using the Baseline Detection Model and the Fusion Model (FM) under different thresholds for prediction probability. The performance of both models is compared based on four key metrics: accuracy, precision, recall, and F-measure, as well as model size and GFLOPs (Giga Floating Point Operations per second).

**Table 9:** Average fire prediction probabilities during occlusion.

Models	Occlusion	Normal
FM Single modality (physical sensing)	0.772	0.923
FM Single modality (video)	0.01	0.917
Base Model	0.333	0.837
FM	0.531	0.97

The Baseline Model demonstrates a relatively strong performance at a threshold of 0.1, achieving an accuracy of 0.842, precision of 0.892, recall of 0.834, and an F-measure of 0.862. However, as the threshold increases, the model’s performance begins to fluctuate. Notably, at a 0.9 threshold, the recall drops significantly to 0.733, even though the accuracy improves to 0.874, and the F-measure decreases to 0.796. This suggests that at higher thresholds, the model becomes more conservative in detecting fires, potentially leading to an increase in false negatives (missed detections).

In comparison, the Fusion Model (FM) outperforms the Baseline Model at all threshold levels. At a 0.1 threshold, the FM achieves an accuracy of 0.932, precision of 0.943, recall of 0.961, and F-measure of 0.952, all of which are considerably higher than the corresponding values of the Baseline Model. As the threshold increases, the FM maintains its strong performance, especially at the 0.9 threshold, where it reaches a recall of 0.988, an accuracy of 0.976, and an F-measure of 0.947. These results indicate that the FM provides more reliable and comprehensive fire detection, particularly when high recall is required to minimize false negatives.

Regarding computational resources, the FM has a slightly larger model size and higher GFLOPs compared to the Baseline Model, with values of 26.2 MB and 12.2 GFLOPs, respectively, compared to the Baseline Model’s 25.04 MB and 11.915 GFLOPs. While these additional computational costs are present, they are justified by the significant improvements in model performance. The FM’s higher recall, lower false negative rate, and overall better performance make it more suitable for high-stakes fire detection applications, where detection reliability is critical.

#### Fire prediction during occlusion

In the occlusion experiment, the camera was covered and none of the video frames acquired contained any objects. When the video camera was occluded or stopped functioning, the fire prediction accuracies of the models that used visual information decreased substantially, as shown in Table 9.

Table 9 compares the fire prediction probabilities of different models under occlusion and normal conditions. The FM Single Modality (Physical Sensing) shows strong performance during occlusion (0.772) and excels under normal conditions (0.923). In contrast, the FM Single Modality (Video) struggles with occlusion (0.01) but performs well under normal conditions (0.917). The Base Model exhibits a clear drop in performance when faced with occlusion, while the combined FM model demonstrates a balanced approach, offering strong performance even under occluded conditions. These results highlight the importance of choosing the appropriate modality or combination of modalities based on the expected conditions, with the combined FM offering the most robust solution overall.

#### Fire predictions at different scenes

The MmodalFire dataset was developed to detect indoor fires in confined spaces. Fire detection sensors are used widely to detect fires stably within different indoor scenes and are not easily constrained by factors such as spatial changes. However, when compared with physical sensing-based fire detection, video-based fire detection is more susceptible to environmental effects, and further consideration is required to address the generalizability of the practical application of video-based fire detection techniques to different scenes.

**Fig. 9:**
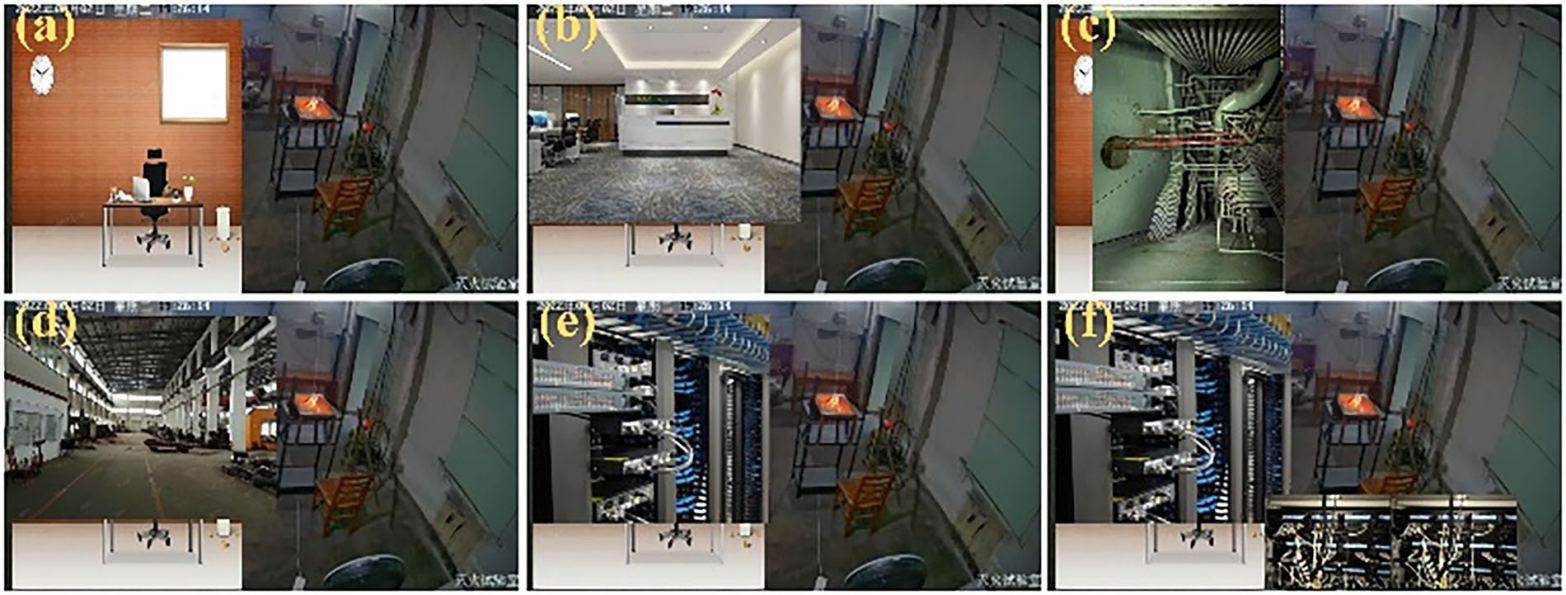
Synthesized video data samples: (**a**–**f**) office, chemical and nuclear power plant, and machine room (without and with cables) image patches when pasted on the left sides of the original video frames.

For the video-based fire detection model shown in the upper branch in Fig. 6(b), we used the “cut and paste” method from the reference^50^ to add image elements from other scenes, including images of offices, chemical and nuclear power plants, and machine rooms with and without cables, to the original video to acquire altered sample videos. Then, for transfer learning, video samples of these altered scenes were added to the original training dataset. This means that, in the original dataset, one-fourth of the original training samples were replaced with these altered video samples, and the model was then trained using the mixed training data. The model’s fire prediction accuracy was subsequently evaluated using all the altered videos. The synthesized video data samples are shown in Fig. 9.

The model’s fire prediction accuracy was evaluated using the original videos and additional videos synthesized from six other scenes. The model’s fire prediction accuracy for the test set and the average fire prediction probabilities for the positive and negative samples are shown in Table 10.

**Table 10:** Test Results Obtained using Videos Synthesized from Six Scenes.

Scenes	Accuracy on test set after transfer learning	Average probability on positive samples	Average probability on negative samples
Original videos	**0.97**	**0.964**	**0.874**
Office 1	**0.953**	0.964	0.714
Office 2	**0.997**	0.824	0.917
Chemical plant	**0.972**	0.935	0.739
Nuclear power plant	**0.927**	0.843	0.813
Machine room	**0.965**	0.922	0.714
Machine room + cable	**0.934**	0.95	0.732

At a threshold of 0.5, the fire prediction accuracy was more than 0.9 for the synthesized video data, thus indicating that some images from different scenes could be used to synthesize videos for use in model training. Note that this experiment only evaluates the generalization of the video modality, as the synthesized videos lack corresponding physical sensing data. Thus, the results cannot be interpreted as evidence of the multimodal (video + physical sensing) model’s robustness in real-world deployment.

However, for the positive and negative samples, the model’s output prediction probabilities decreased slightly when compared with those shown in Table 10, although this is logical because, with the limited synthetic video data that was available, the fire prediction ability of the transfer-learning model declined somewhat. All average fire prediction probabilities were above the 0.5 threshold and the fire detection was still accurate when using the model.

## Usage Notes

To support multimodal fire detection research, we built a multisensor-based platform and established a continuous multimodal dataset called MmodalFire, which is publicly available and can be either fused or used simultaneously with physical sensing-based and video data. MmodalFire includes temperature and smoke density data, radiation data measured at four different wavelengths, and recorded video clips. The data were collected in the same experimental room using the same sensors and camera when operating in different modes for wood, cotton, polyurethane foam, and n-heptane fires, and the negative samples used were dry ice and water mist. The main environmental variables were the airflow and the light intensity, and a total of 65 experimental data groups were included in the dataset, which contains more than 700 min of video footage and supports multisource data analysis during fire detection.

To adjust the fire prediction probability, FM used physical sensing-type data. During gradient maintenance, the neural network’s output was adjusted using the fusion function developed here to calculate the loss value for backpropagation. The FM demonstrated good prediction accuracy. The proposed method may also be used to perform multimodal data fusion. To design a neural network backbone, one or several modalities can be selected, and the other modalities can either be input into the network to extract important physical sensing information or used directly to obtain quantifiable values. The physical sensing information can then be infused into the network backbone to train the model dynamically.

To develop the public MmodalFire multisource fire analysis dataset and identify and monitor fires, physical sensing and video data were integrated and used simultaneously. This dataset will be beneficial for researchers investigating multimodal fire detection using continuous data streams.

For models that are missing a modality or are being deployed in various scenarios, fires can still feasibly be detected using the “cut and paste” method and the reference model.

The rapid development of AI techniques has generated numerous fire detection research opportunities, especially when using object detection, classification, and multimodal analysis methods along with large-scale models. All these techniques fundamentally support the basic framework for specific detection tasks. In future work, we intend to focus on the following aspects. With the help of the dataset established here, we will improve the model, e.g., by fusing the multimodal sensing data with an attention mechanism, for enhanced multimodal fire detection^51^. To evaluate the model’s robustness, we will apply the model to several real detection scenarios. Because actual fires involve combustion of multiple materials, we will investigate the combustion of four fire and two interference materials separately and design further experiments to cover additional fire scenarios.

## Data Availability

The MmodalFire dataset can be downloaded from Figshare^40^.

## Code availability

The baseline neural-network-based fusion models can be found at: https://github.com/SiliconSisyphus/MultiModal-fire-detection.
